# Accessory optic canals and a caroticoclinoid foramen in an infant: considerations for anterior clinoidectomy in the pediatric population

**DOI:** 10.46900/apn.v6i1.233

**Published:** 2024-03-05

**Authors:** Abigail E. Cowher, Matthew J. Zdilla

**Affiliations:** Department of Pathology, Anatomy, and Laboratory Medicine (PALM), West Virginia University School of Medicine, Morgantown, West Virginia, USA

**Keywords:** anatomical variation, anatomy, neurosurgery, pediatrics

## Abstract

**Introduction/Background:**

Anterior clinoidectomy is a routine neurosurgical procedure for the management of paraclinoid aneurysms and tumors in adults; however, it has been performed less extensively in children. The anterior clinoid process of the sphenoid bone is closely related to vital neurovascular structures including the optic nerve, internal carotid artery, and the ophthalmic artery. Therefore, sphenoidal variants, such as the accessory optic canal and caroticoclinoid foramen, pose serious complications in anterior clinoidectomy including potential blindness and death.

**Case presentation:**

This report presents a novel case of concomitant bilateral accessory optic canals and a right-sided caroticoclinoid foramen found within an infantile sphenoid of a 12-month-old black male. The case report documents two co-occurring, clinically relevant variants known to complicate anterior clinoidectomy in an individual from a population that has been underrepresented in the literature.

**Conclusions:**

Caroticoclinoid foramina and accessory optic canals can be mistaken for each other on preoperative imaging, and accessory optic canals can additionally be mistaken for pneumatization of the anterior clinoid process, lesser sphenoidal wing, or optic strut. A high index of suspicion for these anatomic variants on preoperative imaging could enable the prevention of adverse outcomes, including hemorrhaging of the internal carotid artery and/or ophthalmic artery and subsequent blindness or death.

## INTRODUCTION

Anterior clinoidectomy has been performed in the pediatric population for the resection of optic nerve lesions, removal of cavernous malformations, and management of complications due to fibrous dysplasia. [1,2,3] Further, anterior clinoidectomy has been reportedly performed in an infant as young as 18-months. [2]

To facilitate access to the cavernous sinus and posterior orbit and prevent damage to the optic nerve or internal carotid artery during surgery, the optic strut is often detached along with the ACP. [4] However, because of the intimate anatomical relationship between the optic strut and the optic nerve, ophthalmic artery, and internal carotid artery with the optic strut, the risks of anterior clinoidectomy include potential hemorrhage, blindness, and death. [5] Such risks are increased in the presence of local anatomical variations including the caroticoclinoid foramen and the accessory optic canal– bony variations that have been documented to occur unilaterally and bilaterally across races and sexes. [5,6]

Caroticoclinoid foramina form around the internal carotid artery from cornuate bony projections emerging from the anterior clinoid process, middle clinoid process, or both anterior and middle clinoid processes. [6] Thus, CCF encompass the internal carotid artery posteriorly and, accordingly, have been documented to create difficulty in the removal of the ACP. [7,8] The CCF has been identified in the fetal and infantile population at a prevalence of approximately one of every five individuals and has been identified in orbitosphenoid bones from individuals as young as 4-months fetal age. [6]

The accessory optic canal is an anatomical variant which transmits the ophthalmic artery through the optic strut. [5,9] Therefore, a scenario wherein OphA traversed the AOC would predispose damage to the artery and subsequent vision loss during removal of the optic strut. [5,10] Akin to CCF, accessory optic canals have been reported in fetal and infant optic struts, sometimes described as a subtype of so-called metoptic canals. [11,12]

The CCF and AOC have been independently documented in the pediatric population; however, the concomitant presence of the two surgically relevant variants has never been described. This report presents the first documented case of concomitant CCF and AOC in the pediatric population.

## CASE REPORT

**Figure 1-:**
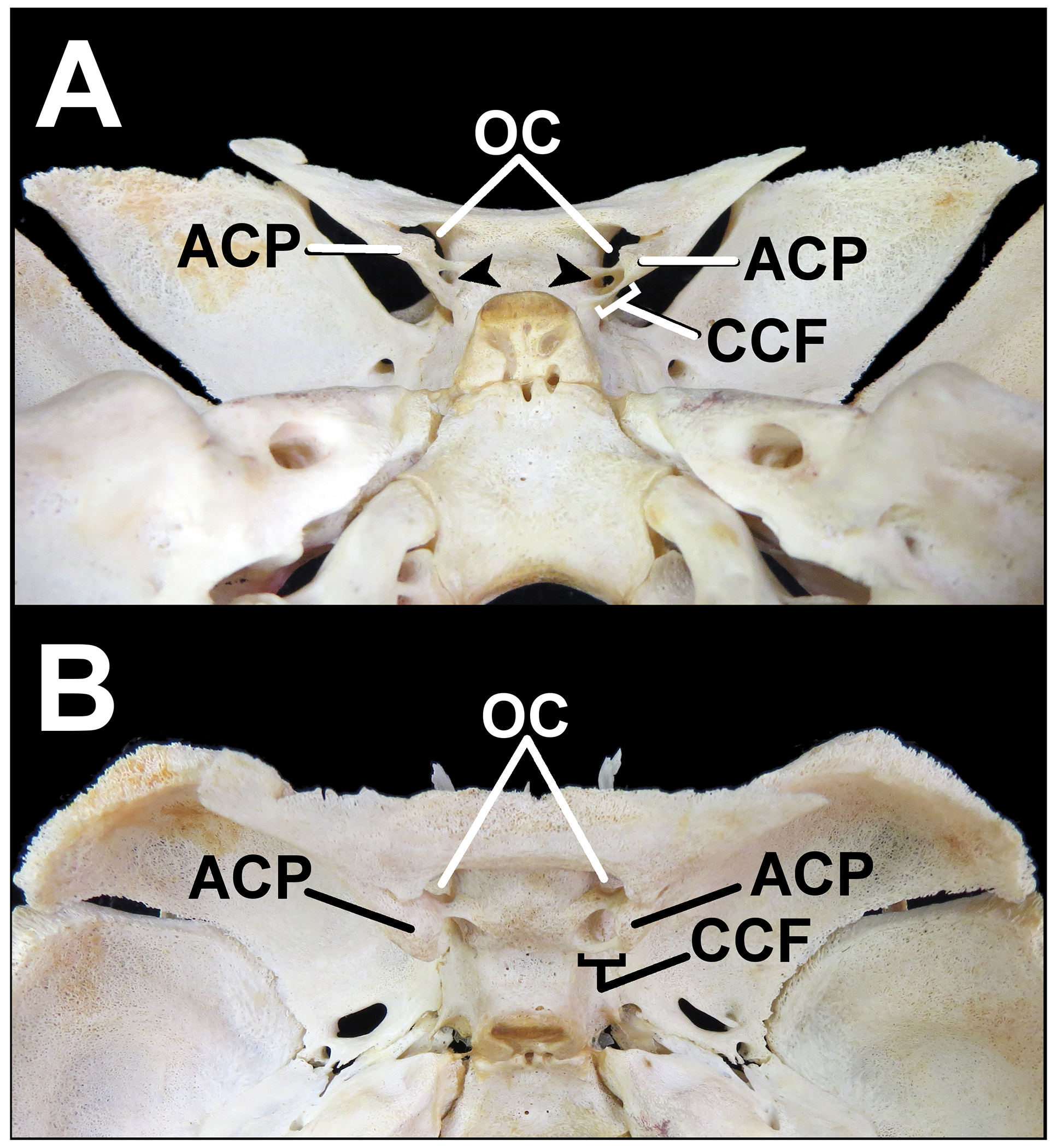
A 12-month-old infantile sphenoid with bilateral accessory optic canals and a right-sided caroticoclinoid foramen. (A) Posterior view. (B) Superior view. (ACP: Anterior clinoid process; CCF: Caroticoclinoid foramen; OC: Optic canal)

**Figure 2-:**
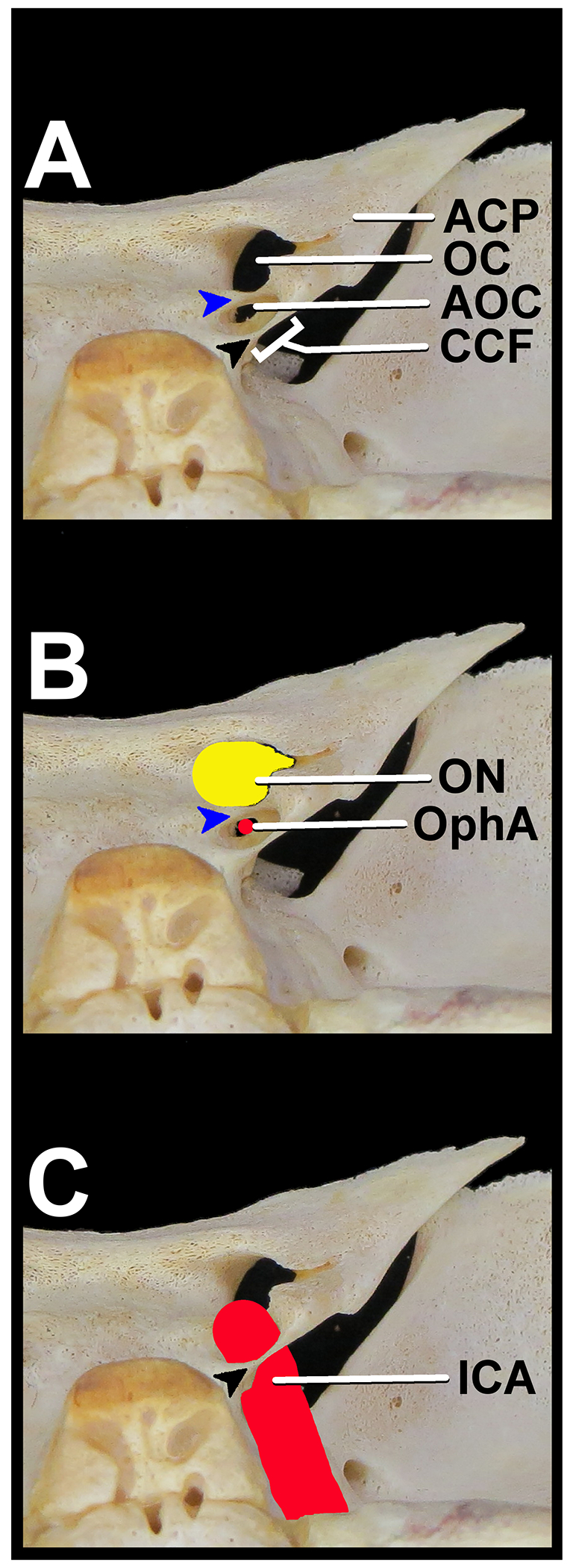
Magnified view of the 12-month-old infantile sphenoid, emphasizing the relationship between the anatomic variants, normal anatomy, and relevant neurovascular structures. (A) View of the variant and typical structures of the lesser sphenoidal wing from the right and posterior. (ACP: Anterior clinoid process; AOC: Accessory optic canal; CCF: Caroticoclinoid foramen; OC: Optic canal; Blue arrow: Bony plate separating the optic canal from the accessory optic canal; Black arrow: Ossified carotico-clinoid ligament, which forms the carotico-clinoid foramen). (B) View of the structures that traverse the optic canal and accessory optic canal from the right and posterior. (OphA: Ophthalmic artery, red; ON: Optic nerve, yellow; Blue arrow: Bony plate separating the optic canal from the accessory optic canal) (C) View of the internal carotid artery traversing the caroticoclinoid foramen before giving rise to the ophthalmic artery, from the right and posterior. (ICA: Internal carotid artery, red; Black arrow: Ossified carotico-clinoid ligament, which forms the carotico-clinoid foramen)

Research was approved through the Department of Anthropology at the Cleveland Museum of Natural History (CMNH). During observation of the Johns Hopkins Fetal Cranial Collection, housed within the CMNH, an infantile sphenoid of a 12-month-old black male was found to have bilateral AOC and a unilateral CCF on the right side (Fig. 1). The right optic strut of the sphenoid bone was both the anterior boundary of the right-sided CCF and the bony structure encompassing the right-sided AOC. A representation of the neurovascular structures regarding their relationship to the AOC and CCF in this specimen can be found in Figure 2.

Measurement of the AOC and CCF was performed by macrophotography and photogrammetry. Digital calipers (Mitutoyo 0-8in (0-203.2mm) ABSOLUTETM digimatic caliper series 500, accuracy +/− 0.001in (0.025mm) were utilized as a fiducial for photogrammetry. The macrophotograph was then taken with a 50× optical zoom camera (Canon PowerShot SX 50 HS, 12.1 Megapixel). The photographs were then analyzed via ImageJ (National Institutes of Health) software by using the set caliper distance as a reference for pixel calibration. The following parameters were assessed: regarding size— major axis, minor axis, area, and perimeter; regarding shape— circularity, roundness, and solidity. [16] Circularity and roundness parameters describe the similarity of a shape to a perfect circle (with perfect being a value of 1.00), whereas solidity describes the convexity of a shape (with perfectly convex having a value of 1.00). [13]

The major axis of the CCF measured 3.6mm, whereas the minor axis measured 3.1mm, and the aspect ratio of 1.15 showcases the relative similarity of these parameters. Continuing, area and perimeter of the CCF were assessed and found to be 8.8mm2 and 11.3mm, respectively. A circularity value of 0.87 and roundness value of 0.87 indicate that the CCF was fairly circular in shape, and a solidity value of 0.97 indicates the CCF was almost completely convex.

The major axis of the right-sided AOC measured 1.6mm, and the minor axis measured 0.9mm. The aspect ratio of 1.79 indicates notable variation between these parameters. Furthermore, area and perimeter of the right-sided AOC were calculated and found to be 1.1mm2 and 5.4mm, respectively. A circularity value of 0.49 and roundness value of 0.56 leads one to infer the right-sided AOC was not very circular in shape. Additionally, a solidity value of 0.91 indicates the right-sided AOC was relatively convex. The optic strut that contained the right-sided AOC was also assessed and was measured to be 2.5mm at its narrowest point and 4.7mm at its widest point. The distance between the midpoint of the superior aspect of the right-sided AOC and the superior border of the optic strut was 1.0mm; the distance between the midpoint of the inferior aspect of the right-sided AOC and the inferior border of the optic strut was 1.3mm.

The major axis of the left-sided AOC measured 1.5mm; the minor axis was 0.8mm, and the aspect ratio of 2.03 displays evident discrepancy between these parameters. Moreover, area and perimeter of the left-sided AOC were determined and found to be 0.9mm2 and 4.6mm, respectively. Circularity was measured and found to be 0.56, whereas the roundness value was 0.49. The aforementioned measurements point to the left-sided AOC not being very circular in shape. Also, a solidity value of 0.94 indicates the left-sided AOC was relatively convex. The optic strut that contained the left-sided AOC was assessed as well and was measured to be 1.8mm at its narrowest point and 4.3mm at its widest point. The distance between the midpoint of the superior aspect of the left-sided AOC and the superior border of the optic strut was 1.1mm; the distance between the midpoint of the inferior aspect of the left-sided AOC and the inferior border of the optic strut was 1.5mm.

## DISCUSSION

This report represents the only documented case of concomitant AOC and CCF in the pediatric population. Though both variations are known to exist independently in the pediatric population, this report establishes that both surgically relevant variations may occur together. Therefore, surgeons performing anterior clinoidectomy must be aware that concomitant AOC and CCF may be found in a patient of any age, in order to anticipate pitfalls in anterior clinoidectomy.

Anterior clinoidectomy is an established and valuable procedure in the adult population; however, the procedure is sparsely reported in the pediatric population. [1,2,3] This disparity in the application of anterior clinoidectomy may be due to the relative lack of literature regarding surgically relevant anatomical variations, including CCF and AOC, in infants and children. Thus, with regard for the appreciation of potential operative pitfalls during anterior clinoidectomy in the pediatric population, this case is especially valuable and may improve procedural decision making.

The clinical implications of the CCF and AOC are many: CCF and AOC can be mistaken for one another on preoperative imaging, and AOC can additionally be mistaken for pneumatization of the anterior clinoid process, lesser sphenoidal wing, or optic strut. [5] Caroticoclinoid foramina have been known to stretch or compress the ICA and alter ICA morphology. [14] Further, incompletely ossified CCF can have bony spurs that cause severe bleeding. [14] Distortion of the ICA by a CCF can also cause ischemia-related sequelae, including headaches. [14]

Within the context of anterior clinoidectomy, the presence of CCF increases the risk of ICA damage, neurological complications, and fatality. [14] The CCF is formed by the ACP, and removing the ACP is crucial to access the cavernous sinus and the clinoid segment of the ICA to treat aneurysms and resect neoplasms in the vicinity. [5] The optic strut is also typically removed during anterior clinoidectomy, both as a precaution and to facilitate enhanced surgical access in the region. [4] The presence of an AOC within the optic strut signals an OphA that is at risk of damage amid strut removal with a subsequent risk of blindness. [4,5]

It was recently demonstrated in a study of adult crania that, in the absence AOCs, optic canals have a larger area, perimeter, and minor axis of a best fit ellipse than the optic canals that occurred alongside AOCs. [15] Thus, optic canal measurement may improve pre-surgical screening for the presence of an AOC. [15] This report documented bilateral presence of AOCs and, therefore, such a side-to-side comparison of optic canals in the presence and absence of AOCs was not feasible. Future studies regarding the potential to utilize the size and shape of the optic canal to identify the AOC in the pediatric population are warranted.

## CONCLUSION

This report establishes the possibility of coexisting AOC and CCF in the pediatric population. Thus, it is imperative that surgeons be aware of the possibility of simultaneous ipsilateral occurrence of the CCF and AOC in infants and children. A high index of suspicion for these anatomic variants on preoperative imaging could enable the prevention of adverse outcomes, including hemorrhaging of the ICA and/or OphA and blindness or death.
